# Microbes

**Published:** 1890-02-01

**Authors:** 


### RECENT RESEARCH.

### MICROBES.

Dr. Witham, of Dorpat, examined a number of cases of granular lids, with the view of deciding whether the cocci found in this affection are the cause. Eight inoculations were performed on healthy human conjunctivæ, but in no case did a granular condition result. This tends to throw a doubt on the theory that microbes have a definite pathogenic relation to the disease. There is indeed a growing feeling, especially in America, that too great latitude has been given to microbes. The presence of such organisms is not questioned, but that there is a definite microbe for every disease is a theory not universally received.

